# Silencing of *dre4* Contributes to Mortality of *Phyllotreta striolata*

**DOI:** 10.3390/insects13111072

**Published:** 2022-11-20

**Authors:** Dongping Chen, Ru Yan, Zhanyi Xu, Jiali Qian, Yinfang Yu, Shunshun Zhu, Huiming Wu, Guonian Zhu, Mengli Chen

**Affiliations:** 1 The Key Laboratory for Quality Improvement of Agricultural Products of Zhejiang Province, College of Advanced Agricultural Sciences, Zhejiang A&F University, Hangzhou 311300, China; 2 Institute of Pesticide and Environmental Toxicology, Zhejiang University, Hangzhou 310030, China; 3 Research and Development Center, NeoAgro Co., Ltd., Hangzhou 310022, China

**Keywords:** *Phyllotreta striolata*, *dre4*, RNAi, mortality, transcriptome analysis

## Abstract

**Simple Summary:**

Biopesticides developed based on RNA interference (RNAi) are specific, efficient and environmentally friendly pesticides for the biological control of pests. *Phyllotreta striolata* is one of the most destructive pests of Cruciferae crops. RNAi is a promising alternative strategy for *Phyllotreta striolata* control, since the larvae of *P. striolata* feed on roots and the adults feed on leaves. However, little information on the lethal effects of RNAi is available for *P. striolata*. In this study, we identified that the *dre4* gene, which plays a significant role in the process of gene transcription, DNA repair and DNA replication, is critical for *P. striolata’s* survival. We found that the silencing of dre4 contributed to the high mortality of *P. striolata* through microinjection and oral delivery. Moreover, we identified that transcripts of multiple gene-related signaling pathways were varied by the silencing of *dre4*, which might have caused the lethality of the *P. striolata*. Overall, our findings indicate that *dre4* could be a fatal RNAi target to develop biopesticides for *P. striolata* management.

**Abstract:**

The striped flea beetle, *Phyllotreta striolata,* is one of the most destructive pests of Cruciferae crops worldwide. RNA interference (RNAi) is a promising alternative strategy for pest biological control, which overcomes the weakness of synthetic insecticides, such as pest resistance, food safety problems and toxicity to non-target insects. The homolog of Spt16/FACT, *dre4* plays a critical role in the process of gene transcription, DNA repair, and DNA replication; however, the effects of *dre4* silencing in *P. striolata* remain elusive. In this study, we cloned and characterized the full-length *dre4* from *P. striolata* and silenced *Psdre4* through microinjection and oral delivery; it was found that the silencing of *dre4* contributed to the high mortality of *P. striolata* in both bioassays. Moreover, 1166 differentially regulated genes were identified after *Psdre4* interference by RNA-seq analysis, which might have been responsible for the lethality. The GO analysis indicated that the differentially regulated genes were classified into three GO functional categories, including biological process, cellular component, and molecular function. The KEGG analysis revealed that these differentially regulated genes are related to apoptosis, autophagy, steroid hormone biosynthesis, cytochrome P450 and other signaling pathways. Our results suggest that *Psdre4* is a fatal RNAi target and has significant potential for the development of RNA pesticides for *P. striolata* management.

## 1. Introduction

The striped flea beetle, *Phyllotreta striolata* (Fabricius) (Coleoptera: Chrysomelidae), an oligophagous pest worldwide, mainly feeds on cruciferous vegetables, such as cabbage, radishes, mustard, etc. The larvae feed on the roots of plants, disturbing the absorption and transmission of nutrients by the roots, thus affecting the growth of plants and eventually leading to plant death. The adults feed on leaves and decrease plant photosynthesis and vegetable quality [1,2,3,4]. The lifespans of larval and adult beetles are 26 to 33 days or 17 to 55 days, respectively, resulting in heavily overlapping generations [5]. Due to the continuous expansion of vegetable cultivation areas and the lack of natural biological factors to control the beetle in the field, *P. striolata* gradually became the most threatening pest to cruciferous vegetables [2,3]. However, the intensive and long-term use of pesticides in beetle interventions has resulted in a rapid and widespread increase in resistance [6]. In addition, insecticides are probably harmful to humans, beneficial insects, fish and birds, since their application in pest control inappropriate, and insecticide residue causes a series of problems for ecosystems and food safety [7,8].

The mechanism of RNA interference (RNAi) is that the type III ribonuclease Dicer digests double-strand RNA (dsRNA) into small siRNAs (siRNA), and siRNAs incorporated into RNA-induced silencing complex (RISC) serve as guides to specifically degrade the homologous mRNA [9,10]. Recently, a highly species-specific and environmentally friendly dsRNA was developed as a useful tool to control pests, indicating that RNAi-based pesticides have significant potential in pest management [11,12]. Currently, scientists focus on developing biopesticides based on RNAi technology to control worldwide pests, including *Diabrotica virgifera virgifera* [13,14], *Leptinotarsa decemlineata* [15], *Henosepilachna vigintioctopunctata* [16,17], *Aphis gossypii* [18], *Myzus persicae* [19], and other pests. Large companies (e.g., Monsanto, Bayer Cropscience, and Syngenta) and start-up companies have commercial interests in RNA applications and attempt to exploit platforms or biotechnology tools [20]. A transgenic maize strain named SmartStax PRO was developed by Dow AgroSciences and Monsanto, which expressed both *Diabrotica*-active Bt and DvSnf7 RNAi traits and reduced rootworm emergence by 80–95% [21]. *P. striolata,* a worldwide pest, is destructive to cruciferous vegetables. Due to their different feeding characteristics, concurrent interventions against both larvae and adults remain an intractable problem. However, the many advantages of RNAi technology, including the easy absorption and conduction of dsRNA in plants, diverse target sites, and high gene-silencing efficiency, might turn RNA pesticides into potential tools with which to control *P. striolata* and other Coleoptera pests [2,10,22,23,24]. However, there are few studies about RNAi in *P. striolata* due to the lack of genome and transcriptome annotation.

The gene *dre4*, a homolog of *spt16* (i.e., a suppressor of Ty16), is a part of the facilitates chromatin transcription (FACT) complex, which participates in the process of gene transcription, DNA repair, and DNA replication [25,26]. FACT has histone chaperone activity and plays an important role in transcriptional extension using chromatin as a template [27,28]. By destabilizing nucleosomes and removing histones, FACT helps RNA polymerase II (RNAPII) pass through nucleosomes smoothly and increases the transcriptional extension of DNA. After RNAPII passes through nucleosomes, histones are represented to the DNA, and nucleosomes are reassembled [28,29]. The FACT complex binds to the six-membered helicase complex, MCM, which is a replicating DNA helicase and is involved in the assembly of the pre-replication complex. In tumor cells, the loss of spt16 led to the downregulation of Bcl2, a negative regulator of apoptosis, thereby inducing tumor cell growth defects [26]. The downregulation of *spt16* expression leads to the activation of DNA damage response. High lethality upon the knockdown of *dre4* has been demonstrated in *Tribolium castaneum*, *Diabrotica virgifera virgifera*, and *Meligethes aeneus* [30]. This work examined the effects of silencing *dre4* in *P. striolata* to explore whether *dre4* is a suitable RNAi target gene for the prevention of *P. striolata*. In addition, regulated transcripts belonging to significant signaling pathways caused by RNAi of *dre4* were identified by transcriptome analysis, which was conducive to the subsequent study of *dre4* as a potential RNAi target gene.

## 2. Materials and Methods

### 2.1. Insect Collection and Rearing

The adult *P. striolata* were collected in Hangzhou, Zhejiang, after which they were fed *Brassica juncea* cv. Bau-Sin plants and maintained at 25 °C ± 1 °C, 75% ± 10% relative humidity (RH), with a photoperiod of 12:12 (light:dark).

### 2.2. RNA Isolation, cDNA Synthesis, and Psdre4 Cloning

Total RNA of adult *P. striolata* was extracted using TRIzol reagent (Takara, Toyoko, Japan) following manufacturer’s instructions. Next, 2 μg of total RNA was used for first-strand cDNA synthesis for gene cloning with Superscript III First-Strand Synthesis System (Invitrogen, Carlsbad, CA, USA), according to manufacturer’s protocol. Specific primers of *Psdre4* were designed based on the nucleotide sequence of *P. striolata* transcriptome. PCR was performed using Phusion^®^ High-Fidelity DNA Polymerase (New England BioLabs, Massachusetts, MA, USA) with the following parameters: 98 °C for 30 s, 35 cycles of 98 °C for 30 s, 55 °C for 20 s and 72 °C for 3 min. The PCR product was cloned into pEASY-Blunt Zero cloning vector (Transgen BioTech, Beijing, China) and verified by sequencing.

### 2.3. Alignment and Phylogenetic Analysis

The *dre4* amino acid sequences from 27 species were downloaded from NCBI database. Multiple sequence alignment was performed using ClustalX 1.83. The neighbor-joining tree was constructed using MEGA 5.0 software with bootstrap value of 1000 replications.

### 2.4. Preparation of the dsRNA

The dsRNA primers flanked with T7-promoter sequences at the 5′ends for *Psdre4* were designed using Primer 5.0 software. The EGFP (enhanced green fluorescent protein, GenBank accession no. U87974) fragment was amplified from the plasmid, pBmEGFPN1, which was kindly provided by Prof. Naiming Zhou from Zhejiang University. The primers of dsEGFP were developed by Peng et al. [31]. The primer sets used for dsRNA synthesis are presented in Table 1. The dsEGFP (414 bp) and dsPsdre4 (514 bp) were synthesized via in vitro transcript using TranscriptAidTM T7 High-Yield Transcription Kit (Ambion, Austin, TX, USA), according to the manufacturer’s protocol. 

### 2.5. Administration of dsRNA by Microinjection

Adult *P. striolata* were injected with 100 nL dsRNA into the hemocoel on the dorsal side of the mid-body by Nanoject II TM injector (Drummond Scientific Company, Broomall, PA, USA) after being anesthetized with CO_2_. Doses of 20 ng, 50 ng, or 100 ng dsPsdre4 were separately injected into adult beetles. Both RNase-free water and 100 ng dsEGFP were used as negative controls. After injection of dsRNA, adults were reared in 150 mL plastic triangular flasks with sponge plugs and fed on leaves of *Brassica juncea* cv. Bau-Sin plants. Three biological replicates were carried out with 10 adults for each dose treatment. Each experiment was repeated at least three times. 

### 2.6. Administration of dsRNA by Oral Delivery

The synthesized dsRNA was sprayed uniformly on both sides of 4 cm × 3 cm leaves of *Brassica juncea* cv. Bau-Sin plants every two days. The treated leaves were used to feed adult beetles after air drying. The concentrations of dsPsdre4 were 200 ng/cm^2^ and 500 ng/cm^2^. RNase-free water or 500 ng/cm^2^ dsEGFP sprayed leaves were set as controls. Three biological replicates were carried out with 10~15 larvae per concentration. The collected data of three independent feeding bioassays were analyzed by SPSS.

### 2.7. Reverse Transcriptase Real-Time Quantitative PCR (RT-qPCR) Analysis

RT-qPCR was carried out on QuantStudio3 Real-Time PCR System (Applied Biosystems, Foster City, CA, USA) using TB Green^®^ PerfectStart Green qPCR SuperMix kit (TransGen, Beijing, China) and gene-specific primers (Table 1). The RT-qPCR was conducted following manufacturer’s protocol. The housekeeping gene *ACT1* was used as internal reference [32]. Relative mRNA levels were normalized to reference gene with the 2^−∆∆Ct^ method [33]. Three biologically independent replicates and three technical replicates were conducted to examine the expression level of Psdre4. 

### 2.8. Transcriptome Analysis after Silencing of Psdre4

To explore effects of silencing of Psdre4, adult *P. striolata* injected with 100 ng dsEGFP or dsPsdre4 were collected and rapidly frozen using liquid nitrogen. Three biological replicates were carried out with 15 adults for each treatment. Total RNA was extracted following the manufacturer’s procedure; the quality and quantity of extracted RNA were examined before Illumina sequencing. The construction of cDNA library and sequencing and analysis of transcriptomic array data were carried out by Personalbio Biotechnology Co., Ltd., (Shanghai, China). Clean reads were spliced using Trinity software to obtain transcripts for subsequent analysis. High-quality sequences were spliced based on the DBG (De Bruijn Graph) splicing principle. The longest transcript of each gene was selected as unigene, which was annotated by NR (NCBI non-redundant protein sequences), GO (Gene Ontology), KEGG (Kyoto Encyclopedia of Genes and Genome), eggNOG (evolutionary genealogy of genes: non-supervised orthologous groups), Swiss-Prot, and Pfam. The transcriptome expression quantitative software, RSEM, was used to align clean reads of each sample to the reference sequence with the transcript sequence as a reference. Next, the number of reads of each sample aligned to each gene was counted and the FPKM value of each gene was calculated. Differential expression analysis was performed using DESeq.

## 3. Results

### 3.1. Cloning and Phylogenetic Analyses of Psdre4 

**Figure 1:**
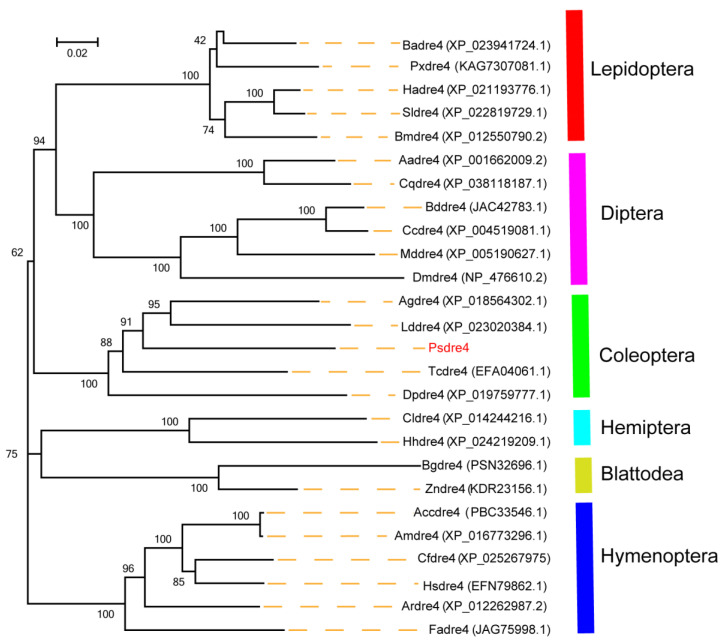
Phylogenetic tree of *dre4* ortholog sequence from 27 insect species. The neighbor-joining phylogenetic tree was generated using the MEGA 5.0 program and bootstrap values from 1000 trials. NCBI GenBank accession numbers are indicated in parentheses. Abbreviations: 1. Lepidoptera (At, *Amyelois transitella*; Ba, *Bicyclus anynana*; Px, *Plutella xylostella*; Ha, *Helicoverpa armigera*; Sl, *Spodoptera litura*; Bm, *Bombyx mori*); 2. Diptera (Aa, *Aedes aegypti*; Cq, *Culex quinquefasciatus*; Bd, *Bactrocera dorsalis*; Cc, *Ceratitis capitata*; Md, *Musca domestica*; Dm, *Drosophila melanogaster*); 3. Coleoptera (Ag, *Anoplophora glabripennis*; Ld, *Leptinotarsa decemlineata*; Ps, *Phyllotreta striolata*; Tc, *Tribolium castaneum*; Dp, *Dendroctonus ponderosae*); 4. Hemiptera (Cl, *Cimex lectularius*; Hh, *Halyomorpha halys*); 5. Blattodea (Bg, *Blattella germanica*; Zn, *Zootermopsis nevadensis*) 6. Hymenoptera (Acc, *Apis cerana cerana*; Am, *Apis mellifera*; Cf, *Camponotus floridanus*; Hs, *Harpegnathos saltator*; Ar, *Athalia rosae*; Fa, *Fopius arisanus*).

**Figure 2:**
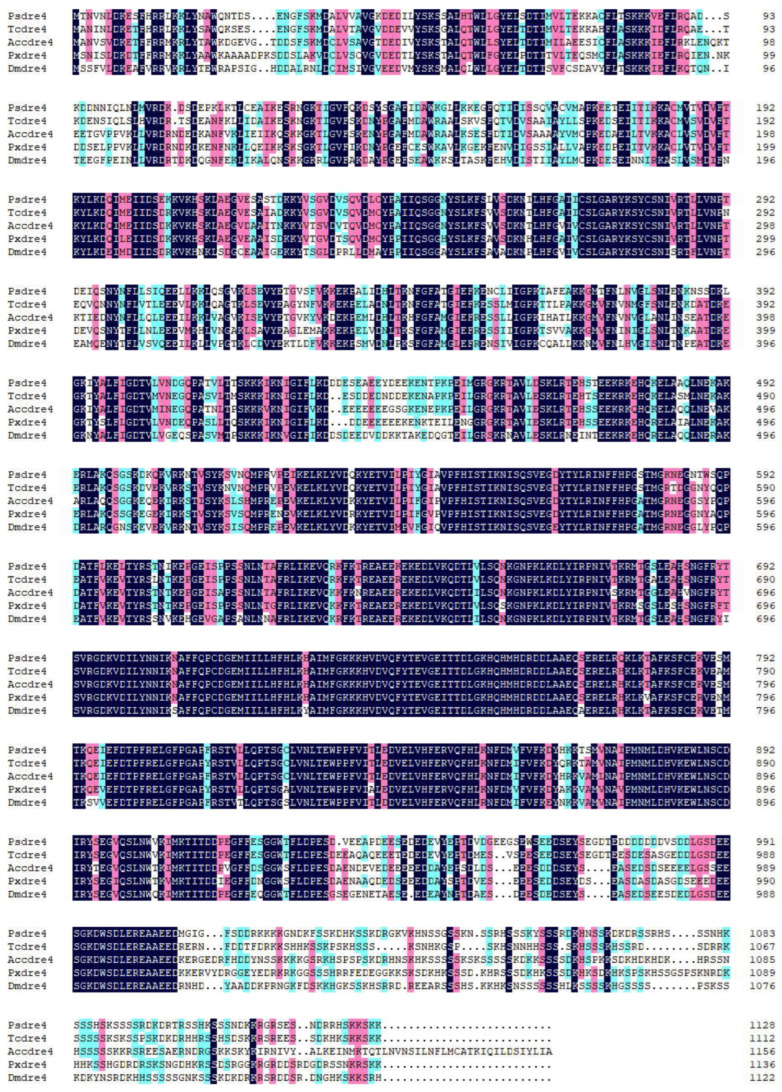
Multiple sequence alignments showing the sequence similarities between Psdre4 and Pxdre4 (*Plutella xylostella*, KAG7307081.1), Dmdre4 (*Drosophila melanogaster*, NP_476610.2), Tcdre4 (*Tribolium castaneum*, EFA04061.1), and Acdre4 (*Apis cerana*, PBC33546.1).

By searching GenBank and the transcriptome data library of *P. striolata*, we obtained the predicated *Psdre4* sequence. Based on the predicted sequence, the *Psdre4* was amplified by PCR, which has a 3387 bp ORF encoding 1129 amino acids. The Psdre4 showed high sequence similarity to the *dre4* homologs of *Drosophila melanogaster*, *Tribolium castaneum*, *Plutella xylostella*, and *Apis cerana* (Figure 1). The percentages of similarity were 65.35% for Dmdre4, 79.45% for Tcdre4, 70.92% for Pxdre4, and 70.32% for Accdre4. Next, we constructed an evolutionary tree of dre4 from 27 species, according to our homology analysis. The neighbor-joining (NJ) phylogenetic tree showed that the *dre4* from the same insect order belonged to a cluster, indicating that the *dre4* was relatively conserved in each order (Figure 2). 

### 3.2. Effect of Silencing Psdre4 on P. striolata Adults by Microinjections

**Figure 3:**
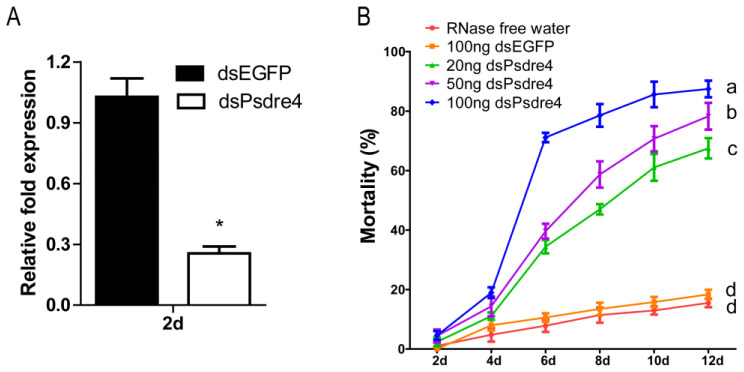
Effects of silencing *Psdre4* on *P. striolata* adults by injection. (**A**). Relative expression levels of *Psdre4* in 2-day beetles after injection of 100 ng dsEGFP or dsPsdre4. Data are plotted as mean ± SEM. Statistical analysis was evaluated using Student’s *t*-test (* *p* < 0.05). (**B**). Percentage mortality of beetles following dsPsdre4 injection. Data are plotted as mean ± SEM. Statistical analysis of percentage mortality on day 12 was determined by using one-way ANOVA followed by Duncan’s multiple-range test. Different letters mean significant differences at *p* < 0.05.

The functional deficiency of *dre4* may have a significant influence on *P. striolata* survival since *dre4* plays a key role in gene transcription extension, DNA replication, and repair. In order to explore the effect of silencing *Psdre4* in *P. striolata*, RNase-free water, 100 ng dsEGFP, and different dilutions of dsPsdre4 were injected into *P. striolata*, respectively. *P. striolata* adults injected with dsEGFP or dsPsdre4 at a dosage of 100 ng after 2 days were used to determine the RNAi efficiency. The results showed that the abundance of *dre4* transcript in dsPsdre4-treated beetles was significantly lower than in beetles injected with dsEGFP (*n* = 6, *p* = 1 × 10^−6^), indicating the designed dsPsdre4 exhibited a suitable interference effect (Figure 3A). Four days after treatment, the mortality rate of *P. striolata* was low, regardless of the treatment concentration of dsPsdre4; however, we noticed that the food intake of the beetles had decreased for 4 days. The mortality rate of *P. striolata* was more than 70% on day 6 after the injection of 100 ng dsPsdre4, which was obviously higher than that of the treatments with 20 or 50 ng. The mortality rates at day 12 caused by 20 ng, 50 ng, and 100 ng dsPsdre4 were 67.51%, 78.28%, and 87.46%, respectively (Figure 3B), indicating that the mortality followed a dose-dependent pattern and disrupted the *Psdre4* gene expression, leading to a high fatality rate of *P. striolata*.

### 3.3. Silencing Effect of Psdre4 in P. striolata Adults through Oral Delivery

**Figure 4:**
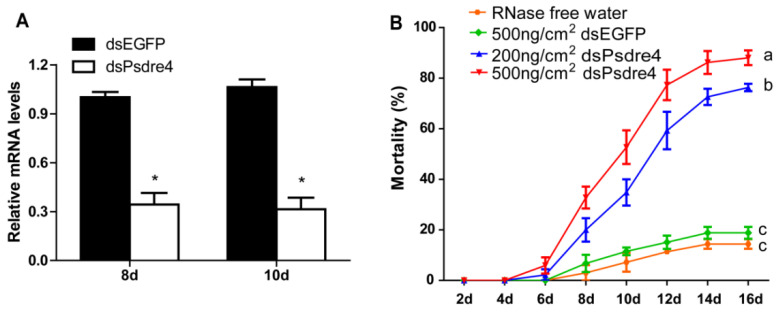
Effects of silencing *Psdre4* on *P. striolata* adults by dsRNA oral delivery. (**A**). Relative expression levels of *Psdre4* in 2-day beetles after treatment with 500 ng/cm^2^ dsEGFP or dsPsdre4. Data are plotted as mean ± SEM. Statistical analysis was evaluated using Student’s *t*-test (* *p* < 0.05). (**B**). The percentage mortality of beetles after dsPsdre4 feeding. Data are plotted as mean ± SEM. Statistical significance between samples on the 16th day was evaluated using one-way ANOVA followed by Duncan’s multiple-range test. Different letters above each bar indicate that the values differed significantly at *p* < 0.05.

In order to verify whether the oral delivery of dsPsdre4 caused a similar effect to the microinjection in *P. striolata*, we conducted a feeding assay with dsPsdre4. The expression levels of *Psdre4* of 8-day and 10-day beetles after feeding with 500 ng/cm^2^ dsPsdre4 were significantly suppressed by 65.83% (*n* = 5, *p* = 2.9 × 10^−6^) and 74.89% (*n* = 5, *p* = 3.9 × 10^−6^), respectively (Figure 4A). The mortality rates when feeding 200 ng/cm^2^ dsPsdre4 and 500 ng/cm^2^ dsPsdre4 were 76.3% and 88.05%, respectively, after 16 days (Figure 4B). These rates were comparable with that of the delivery by microinjection on day 12, suggesting that dsPsdre4 has suitable insecticidal efficacy through both microinjection delivery and oral delivery and could be an excellent RNAi target gene for *P. striolata* control. 

### 3.4. Comparative Transcriptome Analysis

The above results show that the normal function of *dre4* is necessary for *P. striolata* survival, as the silencing of *dre4* leads to high mortality in *P. striolata*. Although the mechanism of lethality induced by the silencing of *dre4* is elusive, to explore which signaling pathways and which fatal genes were affected after interference with *Psdre4*, RNA sequencing was performed on *P. striolata* after injecting 100 ng dsPsdre4 for 4 days.

The cDNA libraries of *P. striolata* were constructed from scratch. In total, 40,193 unigenes were screened from 106,963 transcripts. Furthermore, 40,193 unigenes were compared with proteins in the NCBI NR protein database (E value < 1 × 10^−5^) by using the BLASTX algorithm. As shown in Figure S1A, 52.47% of the unigenes exhibited high homology (E value < 1 × 10^−60^). All the unigenes had BLASTX annotations within the NCBI NR database. The NR species distribution showed that 58% of the unigenes exhibited over 60% identity compared with the other species (Figure S1B). In total, 48.03% of unigenes were identified with six top-hit insect species; *Diabrotica virgifera virgifera* ranked first among the annotated genes (20.98%). The other five top-hit insect species were *Meloidogyne enterolobii* (7.11%), *Halicephalobus* sp. NKZ332 (5.29%), *Gonioctena quinquepunctata* (5.21%), *Leptinotarsa decemlineata* (4.98%), and *Anoplophora glabripennis* (4.46%) (Figure S1C). According to the GO analysis, a total of 20681 unigenes were classified into three GO functional categories, including biological process (BP), cellular component (CC), and molecular function (MF) (Figure S2). A total of 15,539 unigenes were assigned to KEGG pathways (Figure S3).

**Figure 5:**
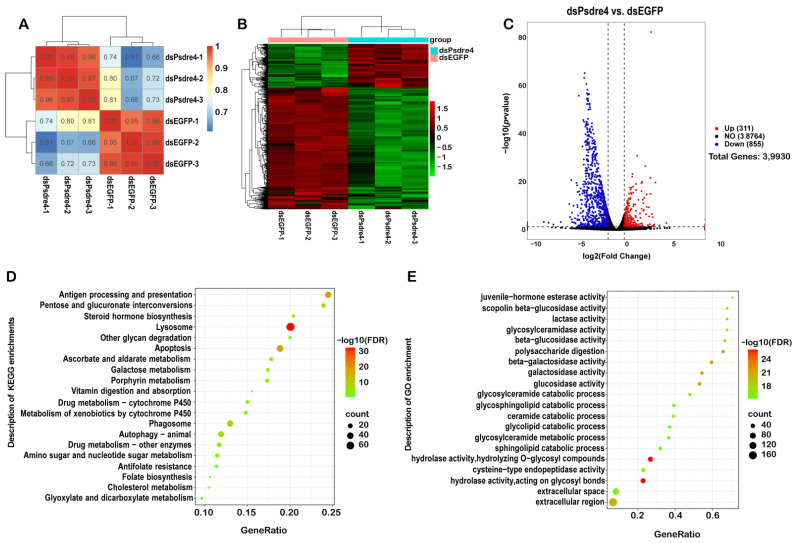
The transcriptomes of *P. striolata* injected with 100ng dsEGFP or 100ng dsPsdre4 were analyzed by RNA-sequencing analysis. (**A**). Pearson’s correlation computation of transcriptome data in all samples. Colors indicate the coefficient values between classes. (**B**). Hierarchical clustering analysis of the transcriptomic data. High-expression genes are shown in red and low-expression genes are shown in green. There were significant differences in the transcriptional levels between dsEGFP-injected groups and dsPsdre4-injected groups. (**C**). The volcano map of transcriptome sequencing results. The abscissa is log_2_ FoldChange, and the ordinate is −log_10_(*p*-value). The two vertical dashed lines in the figure are the two-fold differential expression threshold. The dotted line indicates the threshold of *p*-value = 0.05. Red dots indicate upregulated genes in this group, blue dots indicate downregulated genes, and gray dots indicate non-significantly differentially expressed genes. (**D**). Enrichment results of the KEGG pathway analysis with the highest score and lowest *p*-value for the enrichment score. (**E**). Bubble diagram of GO enrichment result. Twenty enriched terms of GO BP, CC, and MF are presented. In KEGG enrichment results and GO enrichment results, the size of the dot represents the proportion of genes, which is positively associated with the proportion of corresponding enrichment items. The change in color from red to green represents a change in *p*-value from low to high.

RNA-seq was used for the transcriptome analysis. As shown in Figure 5A, the sample correlation within the same group was higher than that between different groups, indicating that the sample repeatability within the group was satisfactory. The results of the transcriptome cluster analysis showed strong differences between the groups (Figure 5B). 

The RNA-seq analysis showed that a total of 1166 significantly differentially expressed genes (|log_2_ FoldChange| > 1, *p*-value < 0.05) were identified in the dsPsdre4 treatment compared with the dsEGFP control. Among the 1166 genes, 311 genes were upregulated, and 855 genes were downregulated. In order to further clarify the particular biological pathways in which these differentially expressed genes participate, thus inducing insect lethality, we attempted to utilize differentially expressed genes for the enrichment analysis in the KEGG signaling pathway and selected the most significant enrichment for demonstration. The knockdown of *dre4* mainly affected apoptosis, autophagy, steroid hormone biosynthesis, cytochrome P450, and other signaling pathways (Figure 5D). A GO functional enrichment analysis was performed on differentially expressed genes, and the most significantly enriched term was selected for display. The most enriched GO terms were related to hydrolase activity, polysaccharide digestion, juvenile hormone esterase activity, etc., which were mainly related to molecular function (Figure 5E). After interference with Psdre4, the expression of the juvenile hormone esterase, growth arrest and DNA damage induction, ATP-dependent translocase, V-type proton ATPase subunit, ATP-binding cassette, and cytochrome P450 were significantly downregulated (Table 2), which might have affected the growth of the insects, even contributing to their deaths.

## 4. Discussion

As a novel, efficient, highly specific, and environmentally friendly biotechnology, RNAi has significant potential for pest control. A previous study cloned and characterized the arginine kinase (AK) gene from *P*. *striolata* and found that silencing the AK gene impaired the beetle’s development, suggesting that AK could be a potential RNAi target [2]. Another study reported that silencing the odorant receptor 1 significantly impaired the host-plant preference of *P. striolata* for cruciferous vegetables [7]. However, few studies focus on the RNAi effect on *P. striolata*, which is hampered by the absence of genome information. In this study, we found that silencing the transcripts of *Psdre4* by injecting or feeding resulted in the death of *P. striolata*, which was identical to previous findings in three Coleoptera pests, *T. castaneum, M*. *aeneus,* and *D. v. virgifera* [30].

Consistent with observations on many other Coleopterans, the oral delivery of dsRNA was highly effective in our study [11,34,35], whereas the effect of the feeding bioassay was slightly less effective than that of the injection bioassay, which may have been related to the instability of dsRNA in the midgut and the environment, since dsRNA is easily degraded by nucleases [9,36,37]. The efficiency of RNAi is mainly driven by the dsRNA delivery into cells. To improve dsRNA delivery and reduce dsRNA degradation, various materials were applied to develop new delivery methods, including cationic core-shell fluorescent nanoparticles, water-soluble cationic dendrimers (nanocarriers), and cationic liposomes, which exhibited suitable penetration and helped to quickly deliver dsRNA to various insect tissues via the oral route [38,39]. Recently, a new nanomaterial, star polycation (SPc), was developed to deliver dsRNA across insect cuticles for efficient gene silencing and pest control [40,41]. The high mortality rates observed in the *P. striolata* feeding bioassay indicate the clear potential of utilizing RNAi as an alternative control method for *P. striolata*. However, as described above, one of the major challenges to the widespread use of RNAi in pest management is the unreliability of dsRNA delivery methods. Therefore, in the future, it is necessary to establish a delivery system to smoothly deliver dsPsdre4 and quickly kill *P. striolata* by modifying or wrapping them with nanomaterials, which would protect dsPsdre4 against degradation by microbes in the environment or the nuclease in the gut content and hemolymph [9,36]. 

As a part of the FACT complex, *dre4* is involved in gene transcription, DNA repair, and DNA replication. Numerous studies have been conducted to research the function of *dre4* in yeast or human tumors [42]; however, little information on *dre4* in insects is available. We identified the full length of *Psdre4* and found that the *dre4* from different species were relatively conserved in each order. Furthermore, we found that the C-terminus of the *dre4* from *D. melanogaster*, *T. castaneum*, *P. xylostella*, *A. cerana,* and *P. striolata* were relatively poorly conserved, which suggests that the C-terminus might confer less significance on the function of *dre4*. 

Because of the function of *dre4*, the transcripts of many significant genes might be influenced by *dre4* deficiency. A total of 1166 significantly differentially expressed genes were detected after the silencing of *Psdre4*, including juvenile hormone esterase (*jhe*), ATP-binding cassette transporter (ABC), and V-type proton ATPase subunit. Juvenile hormone esterase hydrolyzes juvenile hormone, which initiates metamorphosis in many insects and affects their growth, development, longevity, and reproduction [3,43]. After the silencing of *dre4*, the expression of growth arrest and DNA damage-inducible protein was upregulated, which resulted in the triggering of cell apoptosis [44]. The silencing of the ABC transporter significantly increased the mortality of *Bemisia tabaci* [45] and resulted in lethal larval and pupal phenotypes in *Plutella xylostella* [46]. The V-type proton ATPase subunit is a proton pump that combines ATP hydrolysis with proton transport across the membrane, coupling ATP hydrolysis, and proton pumping. The knockdown of the V-type proton ATPase subunit led to the death of *Helicoverpa armigera*, *Myzus Persicae,* and *D. v. virgifer* [34,47]. After the silencing of *dre4*, some important genes responsible for multiple biological processes were significantly downregulated, which further accelerated the deaths of the *P. striolata*. The differentially expressed genes were enriched in some signal transduction pathways, such as apoptosis, autophagy, and the metabolism of xenobiotics by Cytochrome P450. Apoptosis and autophagy affect cell growth and lead to cell death [48]. Cytochrome P450 and hydrolase activity affect detoxification ability, metabolic activity, and disease resistance [45,49,50,51,52]. Thus, the silencing of *Psdre4* affects many important signaling pathways through a series of cascade reactions, which is of great significance for further exploring the function of Psdre4. 

## 5. Conclusions

In conclusion, we amplified and characterized the full length of *dre4* from *P. striolata*. We determined that the silencing of *dre4* contributed to the mortality of *P. striolata*, which might have resulted from the induction of differentially expressed vital genes belonging to certain important signaling pathways through the suppression of the transcripts of *dre4*. Our results suggest that *dre4* is a suitable candidate with which to develop biopesticides based on RNAi technology for *P. striolata* management.

## Figures and Tables

**Table 1:** List of primers used in the study.

Purpose	Gene	Forward Primer Sequence	Reverse Primer Sequence
Full-length ORF PCR	*Psdre4*	CGACTTCTTGTAATGGTGGT	GAGTAACAGTAACAACGCAT
dsRNA synthesis	*Psdre4*	TAATACGACTCACTATAGGGAGACCATACAAGAGGAGTTGCTGAAGAA	TAATACGACTCACTATAGGGAGATTCTCTTCTGTGCTGTGTTCT
	*EGFP*	TAATACGACTCACTATAGGGAAGTTCAGCGTGTCCGGC	TAATACGACTCACTATAGGGCACCTTGATGCCGTTCTTC
qRT-PCR	*Psdre4*	CACGATAGAGACGATTTG	GGTTGGCTGTAGTAATAC
	*PsACT1*	TGTCCCACACTGTACCCATC	CGTGGCCATTTCCTGTTCAA

**Table 2:** Several significantly differently expressed genes associated with survival.

ID	Gene Name	Fold Change (dsPsdre4/dsEGFP)	*p*-Value	padj
TRINITY_DN5071_c0_g1	ATP-dependent translocase ABCB1	0.166	6.37548 × 10^−35^	3.22 × 10^−32^
TRINITY_DN495_c0_g1	ATP-binding cassette sub-family C member 4	0.390	7.31589 × 10^−12^	7.62724 × 10^−10^
TRINITY_DN5071_c0_g1	ATP-dependent translocase	0.166	6.37548 × 10^−35^	3.22244 × 10^−32^
TRINITY_DN5231_c0_g1	V-type proton ATPase subunit G	0.450	3.36152 × 10^−9^	2.6632 × 10^−7^
TRINITY_DN5528_c0_g1	V-type proton ATPase catalytic subunit A	0.340	1.2124 × 10^−15^	1.7733 × 10^−13^
TRINITY_DN7561_c0_g1	V-type proton ATPase subunit H	0.454	3.50111 × 10^−9^	2.76283 × 10^−7^
TRINITY_DN1747_c2_g1	V-type proton ATPase 16 kDa proteolipid subunit	0.428	1.39512 × 10^−10^	1.29552 × 10^−8^
TRINITY_DN3943_c0_g1	Cytochrome P450 4C1	0.410	2.34671 × 10^−8^	1.69447 × 10^−6^
TRINITY_DN9443_c0_g1	Probable cytochrome P450 4d14	0.163	2.37721 × 10^−36^	1.33693 × 10^−33^
TRINITY_DN9990_c0_g1	Probable cytochrome P450 6a14	0.068	5.18681 × 10^−66^	1.03555 × 10^−61^
TRINITY_DN2946_c0_g1	Growth arrest and DNA damage-inducible protein	3.704	2.79063 × 10^−18^	5.13501 × 10^−16^
TRINITY_DN3124_c0_g1	Juvenile hormone esterase	0.036	2.73178 × 10^−19^	5.29514 × 10^−17^
TRINITY_DN9349_c0_g1	*dre4* (FACT complex subunit spt16)	0.206	5.36373 × 10^−25^	1.54082 × 10^−22^

## Data Availability

Data is contained within the article and supplementary material.

## Supplementary Materials

The following supporting information can be downloaded at: https://www.mdpi.com/article/10.3390/insects13111072/s1, Figure S1: Homology analysis of *P. striolata* unigenes, Figure S2: Gene ontology (GO) assignment of *P. striolata* unigenes, Figure S3: Kyoto Encyclopedia of Genes and Genomes (KEGG) classification of *P. striolata* unigene.
