# Supplementary material for: Silencing of dre4 Contributes to Mortality of Phyllotreta striolata
Source: Insects. 2022 Nov 20;13(11):1072. doi: 10.3390/insects13111072 (PMC9696999; doi:10.3390/insects13111072)
Supplement: Supplementary file 1 [file insects-13-01072-s001.zip › insects-2006702-supplementary.pdf]

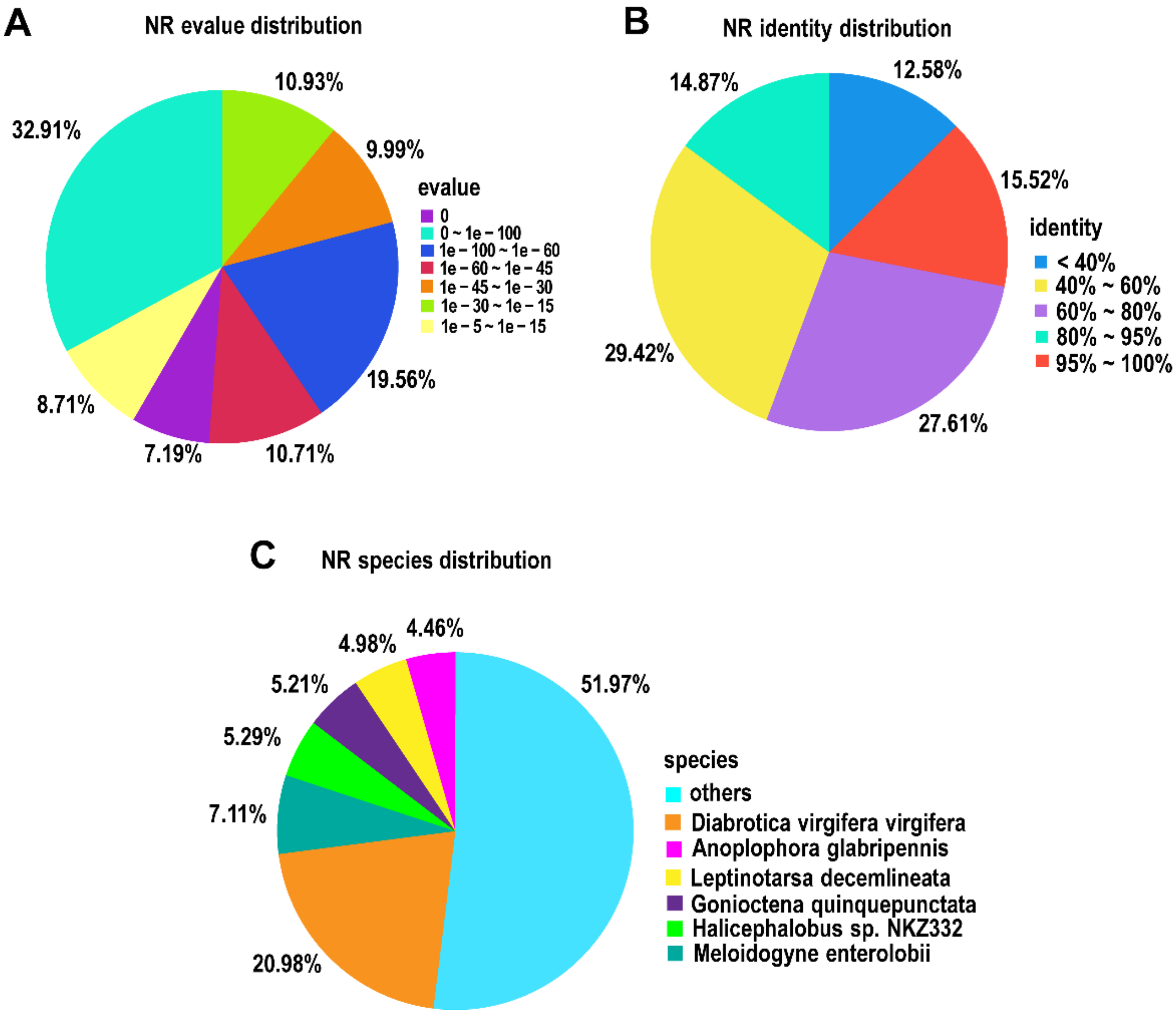


**Figure S1.** Homology analysis of *P. striolata* unigenes. (A) NR-value distribution. (B) NR-identity distribution. (C) NR-species distribution.

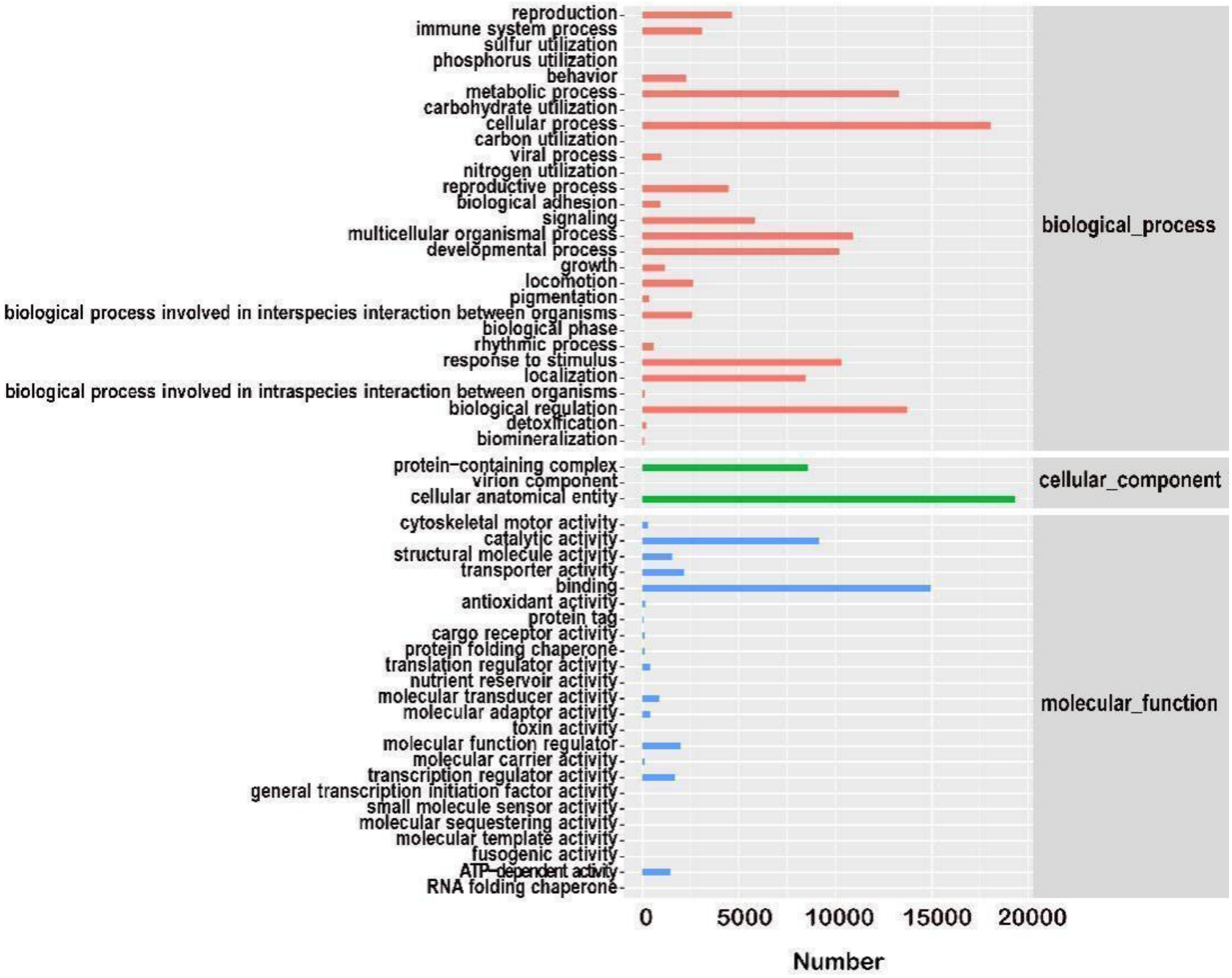


**Figure S2.** Gene-ontology (GO) assignment of *P. striolata* unigenes. Eggnog-mapper software was used to complete GO annotation and the default parameters of EggNog-mapper were used for annotation. The GO-annotation results corresponded to GOTerm and the number of annotated genes in the second-level classification was counted. There were three categories in the GO analysis: biological process (BP), cellular component (CC) and molecular function (MF).

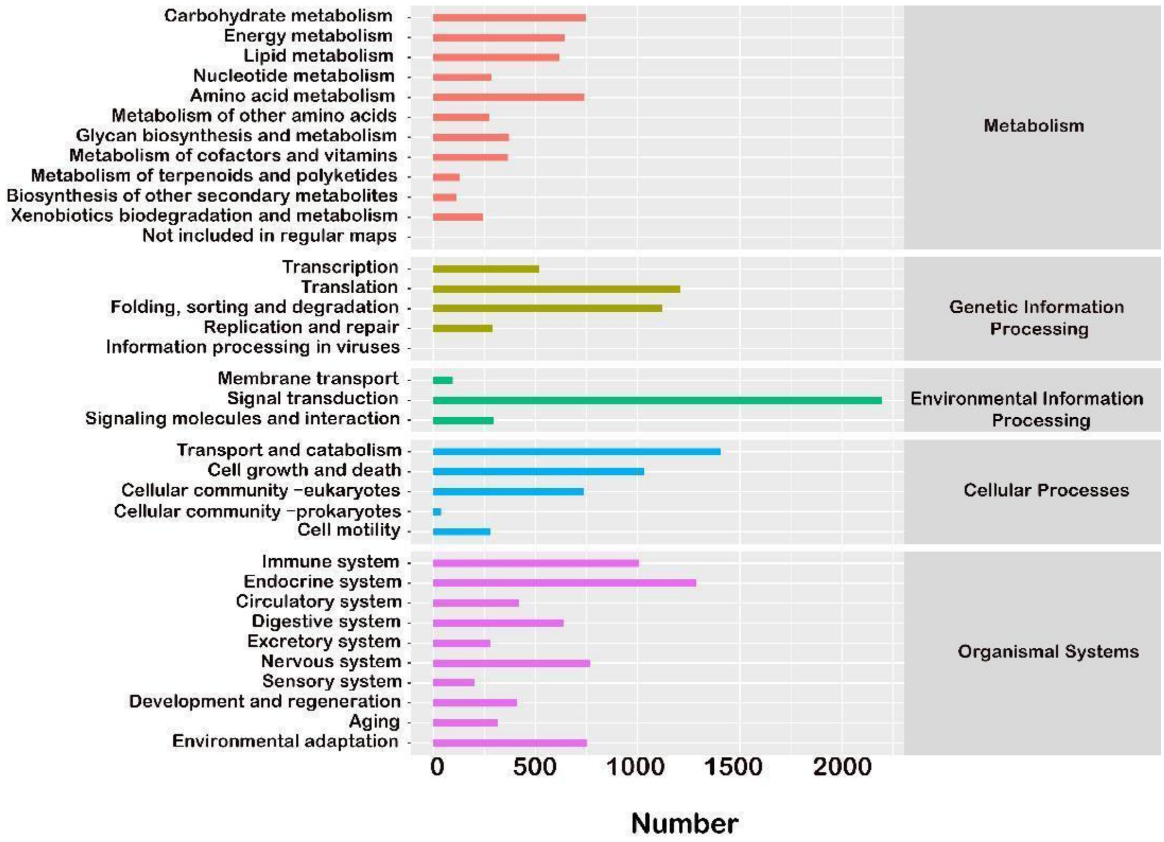


**Figure S3.** Kyoto Encyclopedia of Genes and Genomes (KEGG) classification of *P. striolata* unigene. KO and pathway annotations were mainly completed by KOBAS annotation system (http://kobas.cbi.pku.edu.cn/.). Gene sets selected large groups of species (animals, plants, etc.). KO was mapped to the corresponding KEGG pathway after KO annotation was completed. KEGG classification included five main categories: cellular processes, environmental-information processing, genetic-information processing, metabolism and organism systems.
